# Successes and Barriers of Health Information Exchange Participation Across Hospitals in South Carolina From 2014 to 2020: Longitudinal Observational Study

**DOI:** 10.2196/40959

**Published:** 2023-09-28

**Authors:** Zhong Li, Melinda A Merrell, Jan M Eberth, Dezhi Wu, Peiyin Hung

**Affiliations:** 1 Department of Public Administration School of Health Policy and Management Nanjing Medical University Nanjing China; 2 Department of Health Services Policy and Management Arnold School of Public Health University of South Carolina Columbia, SC United States; 3 Rural and Minority Health Research Center Arnold School of Public Health University of South Carolina Columbia, SC United States; 4 Department of Health Management and Policy Drexel University Philadelphia, PA United States; 5 Department of Integrated Information Technology College of Engineering and Computing University of South Carolina Columbia, SC United States

**Keywords:** health information exchange, electronic health records, interoperability, meaningful use, hospital

## Abstract

**Background:**

The 2009 Health Information Technology for Economic and Clinical Health Act sets three stages of Meaningful Use requirements for the electronic health records incentive program. Health information exchange (HIE) technologies are critical in the meaningful use of electronic health records to support patient care coordination. However, HIE use trends and barriers remain unclear across hospitals in South Carolina (SC), a state with the earliest HIE implementation.

**Objective:**

This study aims to explore changes in the proportion of HIE participation and factors associated with HIE participation, and barriers to exchange and interoperability across SC hospitals.

**Methods:**

This study derived data from a longitudinal data set of the 2014-2020 American Hospital Association Information Technology Supplement for 69 SC hospitals. The primary outcome was whether a hospital participated in HIE in a year. A cross-sectional multivariable logistic regression model, clustered at the hospital level and weighted by bed size, was used to identify factors associated with HIE participation. The second outcome was barriers to sending, receiving, or finding patient health information to or from other organizations or hospital systems. The frequency of hospitals reporting each barrier related to exchange and interoperability were then calculated.

**Results:**

Hospitals in SC have been increasingly participating in HIE, improving from 43% (24/56) in 2014 to 82% (54/66) in 2020. After controlling for other hospital factors, teaching hospitals (adjusted odds ratio [AOR] 3.7, 95% CI 1.0-13.3), system-affiliated hospitals (AOR 6.6, 95% CI 3.2-13.7), and rural referral hospitals (AOR 8.0, 95% CI 1.2-53.4) had higher odds to participate in HIE than their counterparts, whereas critical access hospitals (AOR 0.1, 95% CI 0.02-0.6) were less likely to participate in HIE than their counterparts reimbursed by the prospective payment system. Hospitals with greater ratios of Medicare or Medicaid inpatient days to total inpatient days also reported higher odds of HIE participation. Despite the majority of hospitals reporting HIE participation in 2020, barriers to exchange and interoperability remained, including lack of provider contacts (27/40, 68%), difficulty in finding patient health information (27/40, 68%), adapting different vendor platforms (26/40, 65%), difficulty matching or identifying same patients between systems (23/40, 58%), and providers that do not typically exchange patient data (23/40, 58%).

**Conclusions:**

HIE participation has been widely adopted in SC hospitals. Our findings highlight the need to incentivize optimization of HIE and seamless information exchange by facilitating and implementing standardization of health information across various HIE systems and by addressing other technical issues, including providing providers’ addresses and training HIE stakeholders to find relevant information. Policies and efforts should include more collaboration with vendors to reduce platform compatibility issues and more user engagement and technical training and support to facilitate effective, accurate, and efficient exchange of provider contacts and patient health information.

## Introduction

Health information exchange (HIE) has great potential to support patient transitions and achieve substantial financial and societal benefits across the fragmented United States health care system [1-3]. According to the 2009 Health Information Technology for Economic and Clinical Health (HITECH) Act, there are three stages of Meaningful Use. The goal of Stage 1 of Meaningful Use from 2009 to 2010 was to establish the US federal government’s Meaningful Use incentive programs and to motivate health care professionals and institutions to capture, protect, and electronically store data, thus promoting wide adoptions of electronic health records (EHRs) [4]. Stage 2 of Meaningful Use—which began in 2014—emphasized the use and documentation of advanced clinical processes that guided the information exchange between providers and patients and between providers in the same practice to improve treatment adherence and care coordination. In 2015, the Centers for Medicare & Medicaid Services launched Stage 3 of the Meaningful Use requirements for the EHR Incentive Program. In Stage 3, eligible hospitals must demonstrate the interoperability of EHR systems in different practices with a focus on improving patient outcomes [5], such as improved coordination and efficiency of care by reducing the replication of health care services [6,7]. Previous studies have suggested that HIE implementation can also improve quality of care and holds promise to help achieve the goals of other policies, such as the Hospital Readmission Reduction Program [8,9].

Given the potential benefits, the Centers for Medicare & Medicaid finalized a rule to promote HIE and set exchanging all “necessary health information,” including courses of illness, treatment, and discharge goals, with health care providers at the next level of care as a condition of participation in Medicare [10,11]. Although primary care providers strongly agree that meeting the Stage 3 care coordination criteria would improve patients’ treatment [12], small, rural, and critical access hospitals (CAHs) were less likely to participate in national networks and state, regional, or local health information organizations (HIOs) than other hospitals as of 2018 [13]. Hospitals having a larger market share or those in less competitive markets had a greater probability of HIE participation [14], and nonprofit and publicly owned hospitals were more likely to participate in HIE than for-profit hospitals [15]. Moreover, larger hospitals were more likely to exchange health information internally than with outside hospitals [16]. However, despite the increasing proportion of hospital engagement, patient health information was not exchanged as needed [17]. Emergency department physicians reported that HIE sometimes disrupted their workload [18]. At the organization level, the rates of HIE upon transfer from psychiatric units lagged that from general medicine or surgery hospitals reported as of 2016 [11]. CAHs have been struggling to keep up with other advanced functions, even when these hospitals had EHRs as of 2018 [19], and physicians in solo practices and nonprimary care specialties also lagged [20]. Besides substantially poorer health care infrastructure [21,22], rural hospitals were least likely to have patient engagement capabilities or clinical information available electronically from outside providers [23]. Difficulties in sending and receiving health information and complex workflows are still the main barriers [12]. Previous research pointed out that developers need to work with health care providers to ensure HIE tools are integrated into existing workflows [24]. Costs of electronic interface development might also be a key barrier to fully integrated HIEs [25].

In South Carolina (SC), state-level efforts to encourage HIE were funded by the US Department of Health and Human Services during this time. Building upon existing statewide data infrastructure, SC became an early adopter of HIE starting in 2008 [26]. However, adverse effects of privacy regulation on the successful implementation and use of HIE also raised wide concerns [3], presenting a crucial obstacle to facilitating the exchange of health information between hospitals and across different health systems or distinct physician practices [27]. Because data on the implementation of HIE and barriers to exchange and interoperability of patient health information across hospitals in SC remains unknown, we pursued this study to provide insight for the state government to enact purposive policies and intensive efforts to promote wider participation in and use of HIE. The anticipated benefits gained from HIE come from the realized exchange and use of patient health information across health care providers; thus, whether these health information technologies are run in a supportive environment raised concerns about the barriers SC hospitals face. Previous studies found that technical and cost issues and privacy and security concerns could hinder HIE implementation during the process of HIE expansion [28,29], even with an increasing adoption of EHRs in the United States [30]. As qualification to participate in HIE does not necessarily lead to the use of HIEs [25], identifying and assessing related obstacles may inform policy efforts to address health care providers’ concerns about their HIE use for improved health outcomes when most hospitals have met the criteria of Meaningful Use Stage 3 [27]. As an early adopter of HIE [26], SC, which contains some very rural areas, may have faced unique challenges that might be insightful for the adoption and use of HIE across hospitals and other health care facilities in other states or regions with similar settings. Therefore, using the most recent available survey data, we aimed to explore changes in the proportion of HIE participation and associated factors between 2014 and 2020 and barriers to interoperability as of 2020 across SC hospitals to inform policy makers and providers on how to enact additional policy interventions to ensure HIE adoption and use for a patient-centric health care system.

## Methods

### Study Design

We conducted a retrospective, longitudinal analysis of 69 individual hospitals in SC using the American Hospital Association (AHA) Annual Surveys IT Supplement from 2014 to 2020. This survey was sent to the chief executive officer of every hospital in the United States for completion by themselves or by the most knowledgeable person in each hospital. The AHA Annual Survey included questions about organization structure, technology adoption, and other topics about service provision. The AHA Annual Survey IT Supplement database collects data on facility-level adoption and implementation of the US Department of Health and Human Services Promoting Interoperability initiative, including computerized system capabilities, patient engagement, HIE, barriers to HIE and interoperability, and other factors [31]. The response rates of the AHA IT survey among SC hospitals ranged from 44.9% in 2014 to 59.4% in 2020. The primary question was whether a hospital participated in local HIE activities in a year. According to the AHA Annual Survey, HIE involvement was assessed by indicating the level of participation in a state, regional, and/or local HIE or HIO. Using historical responses, we were able to carry forward and impute the missing values of 14 hospitals based on the following scenarios:

1. Hospitals that responded as having participated in HIE in the previous and subsequent years but did not respond to the survey in a given year were coded as participants.
2. Hospitals reporting no participation in the previous and subsequent years but did not respond to the survey in a given year were coded as nonparticipants.
3. Hospitals that reported no local HIE/HIO or no participation in HIE/HIO in a given year but did not respond in the previous year were coded as nonparticipants in the previous year. For example, if a hospital did not participate in HIE in 2015 and did not report whether they participated in 2014, the hospital would be coded as nonparticipants in 2014.
4. Hospitals that reported having participated in HIE/HIO in the previous year but did not respond in a given year were coded as nonparticipants.

The final analytic data set included 69 unique hospitals, but varied by year, ranging from 56 hospitals in 2014 to 66 hospitals in 2020. In 2014, of the 56 hospitals, only 27 responded on barriers to send or receive patient health information and other barriers related to exchanging patient health information. In 2020, of the 66 hospitals, 40 responded on barriers to send or receive patient health information and other barriers related to exchanging patient health information.

### Variables

The primary independent variable was hospital location by rurality, categorized by Rural-Urban Commuting Area codes into urban (primary codes: 1-3) or rural (4-10). We also derived the following factors from the AHA Annual Surveys: number of beds staffed (1, <100 beds; 2, 100-299 beds; 3, >300 beds), teaching status, ownership (public nonfederal, private for-profit, or private nonprofit hospitals), system-affiliated hospitals (yes or no), primary services code (general or specialty hospitals), Medicare payment scheme (prospective payment system, CAHs, rural referral hospitals or sole community hospitals), ratio of Medicare inpatient days to total inpatient days, and ratio of Medicaid inpatient days to total inpatient days. Furthermore, we set survey year as covariate to examine annual linear trends in the proportion of HIE adoption across SC hospitals.

The second outcome variable was barriers to send, receive, or find patient health information to or from other organizations or hospital systems, which was not imputed because the barriers can change over time. To capture the main barriers to exchange and interoperability of patient health information, each hospital was asked, “Which of the following issues has your hospital experienced when trying to electronically (not eFax) send, receive or find (query) patient health information to/from other organizations or hospital systems?” Per the Healthcare Information and Management System Society, interoperability is the ability of different information systems, devices, and applications to access, exchange, integrate, and cooperatively use data, while exchange is the ability to send or to receive information but not necessary to integrate and harmonize data for further use [32].

### Statistical Analysis

We first compared hospital characteristics by the status of HIE participation and presented geographic distributions of HIE participation across SC hospitals in 2014 and 2020. We then conducted chi-square tests to compare hospital characteristics by HIE participation status. Cross-sectional multivariable logistic regression models clustered at the hospital level to account for repeated observations and weighted by bed size were used to identify factors associated with HIE participation across hospitals. Additionally, we calculated the frequency of hospitals reporting each barrier related to electronically sending or receiving patient health information or other financial or technical barriers to exchanging patient health information. All analyses were conducted using Stata 15.0 (StataCorp LLC).

### Ethical Considerations

The University of South Carolina Institutional Review Board exempted this study protocol.

## Results

### Proportion of Hospitals With HIE Technologies

**Figure 1:**
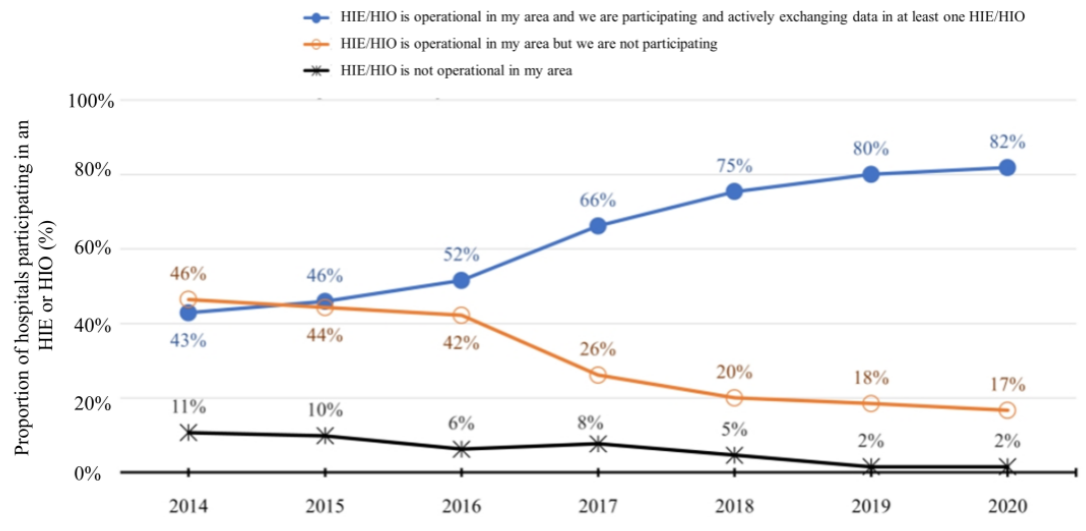
Proportion of hospitals with health information exchange technologies from 2014 to 2020. The proportion of hospitals participating in a health information exchange HIE or HIO increased over time (*P*<.001). Results stratified by hospital location can be found in Multimedia Appendix 1. HIE: health information exchange; HIO: health information organization.

**Figure 2:**
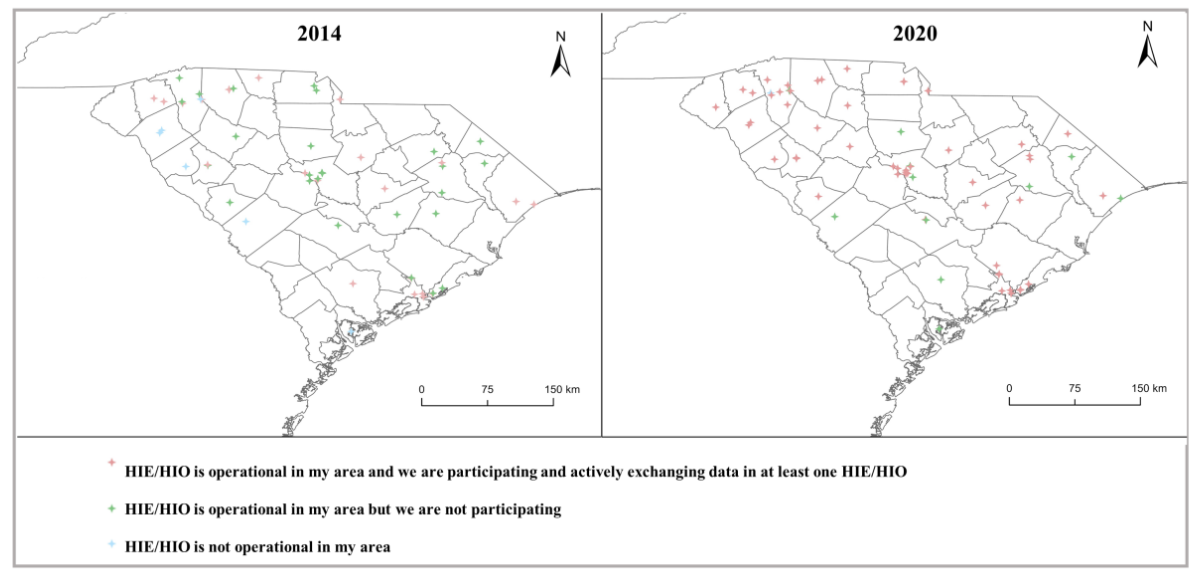
Geographic distribution of HIE participation across South Carolina hospitals in 2014 and 2020. HIE: health information exchange; HIO: health information organization.

In 2014, 24 of 56 (43%) hospitals reported participating in HIE. Private for-profit hospitals, nonteaching hospitals, small hospitals (<100 beds), non–system-affiliated hospitals, and specialty hospitals were less likely to participate in HIE than their peers. In 2020, 54 of 66 (82%) hospitals reported participating in HIE (Table 1). In 2020, 77% (13/17) of rural hospitals reported participating in HIE, which was comparable to the 84% (41/49) participation rate among urban hospitals. Between 2014 and 2020, there was an increasing trend in HIE participation among sample hospitals (Figure 1). The geographic distribution of these hospitals per their self-reported HIE participation status is illustrated in Figure 2. In 2020, hospitals in the Upstate region mostly participated in HIE or HIO, whereas hospitals in many rural counties did not participate despite the HIE network availability in their local areas. The proportions of rural and urban hospitals with HIE or HIO participation increased at similar rates between 2014 to 2020 (Multimedia Appendix 1).

**Table 1:** Hospital characteristics by health information exchange participation status in 2014 and 2020.^a^

Characteristics		2014 (n=56)			2020 (n=66)		
		Yes, n (%)	No, n (%)	*P* value	Yes, n (%)	No, n (%)	*P* value
All respondent hospitals		24 (43)	32 (57)	N/A^b^	54 (82)	12 (19)	N/A
**Rurality**				.10			.51
	Rural	3 (23)	10 (77)		13 (77)	4 (24)	
	Urban	21 (49)	22 (51)		41 (84)	8 (16)	
**Hospital ownership**				.37			.19
	Public nonfederal	7 (39)	11 (61)		16 (73)	6 (27)	
	Private nonprofit	11 (55)	9 (45)		24 (92)	2 (8)	
	Private for-profit	6 (33)	12 (67)		14 (78)	4 (22)	
**Teaching hospitals**				.02			.43
	Yes	3 (100)	0 (0)		3 (100)	0 (0)	
	No	21 (40)	32 (60)		51 (81)	12 (19)	
**Hospital bed size**				.02			.04
	<100 beds	8 (28)	21 (72)		34 (90)	4 (11)	
	100-299 beds	7 (47)	8 (53)		9 (60)	6 (40)	
	>300 beds	9 (75)	3 (25)		11 (85)	2 (15)	
**Affiliation to health system**				.12			.06
	Yes	19 (49)	20 (51)		45 (87)	7 (14)	
	No	5 (29)	12 (71)		9 (64)	5 (36)	
**Specialty hospitals**				.06			.95
	Yes	3 (21)	11 (79)		14 (82)	3 (18)	
	No	21 (50)	21 (50)		40 (82)	9 (18)	
**Medicare payment scheme**				.05			.88
	Critical access hospitals	0 (0)	4 (100)		3 (75)	1 (25)	
	Rural referral hospitals	2 (100)	0 (0)		6 (86)	1 (14)	
	Sole community hospitals	1 (50)	1 (50)		3 (75)	1 (25)	
**Ratio of Medicare inpatient days to total inpatient days**				.57			.10
	<47.0%	8 (38)	13 (62)		17 (74)	6 (26)	
	47.0%-58.8%	10 (53)	9 (47)		15 (75)	5 (25)	
	>58.8%	6 (38)	10 (63)		22 (96)	1 (4)	
**Ratio of Medicaid inpatient days to total inpatient days**				.21			.79
	<8.0%	6 (30)	14 (70)		15 (79)	4 (21)	
	8.0%-14.4%	11 (58)	8 (42)		19 (86)	3 (14)	
	>14.4%	7 (41)	10 (59)		20 (80)	5 (20)	

^a^Data are presented with consideration of missing values for the category variables.

^b^N/A: not applicable.

### Barriers to Exchange and Interoperability Across SC Hospitals

**Figure 3:**
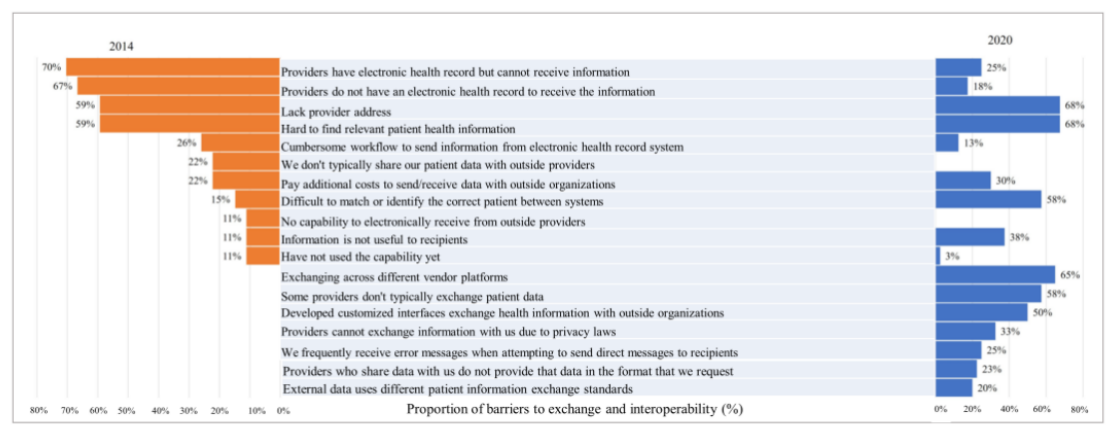
Barriers to exchange and interoperability across South Carolina hospitals in 2014 (n=27) and 2020 (n=40). EHR: electronic health record.

As shown in Figure 3, among hospital respondents, the leading barriers to HIE in 2020 were difficulties in locating the contact information of the providers to send patient health information (27/40, 68%) and finding relevant patient health information (27/40, 68%), followed by several technical and systemic issues, including exchanging across different vendor platforms (26/40, 65%), difficulty matching or identifying the correct patient between systems (23/40, 58%), and providers that do not typically exchange patient data (23/40, 58%). Additionally, 33% (13/40) of sample hospitals reported that providers could not exchange information due to privacy laws as barriers to exchange and interoperability. In 2014, the most commonly reported barriers to exchange and interoperability were providers having EHRs but being unable to receive information (19/27, 70%), providers lacking an EHR to receive information (18/27, 67%), difficulty locating providers' address (16/27, 59%), difficulties in finding relevant patient health information (16/27, 59%), and cumbersome workflow to send information from the EHR system (7/27, 26%).

### Factors Associated With Hospitals’ HIE Participation

**Figure 4:**
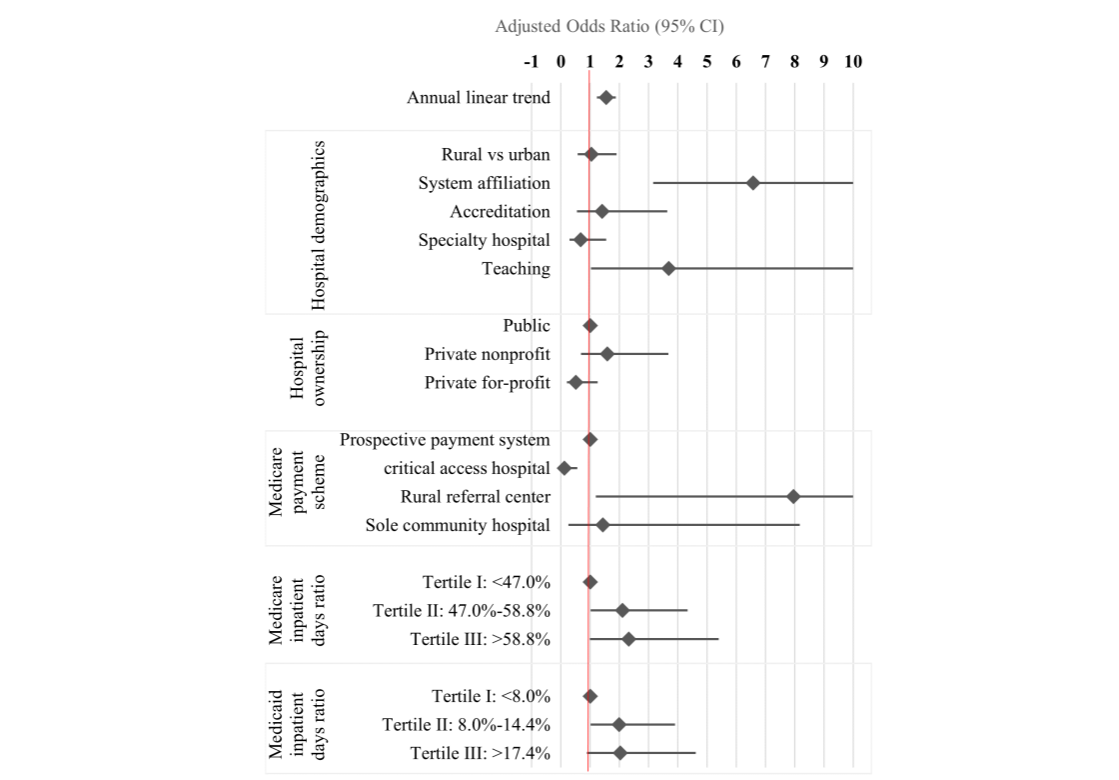
Multivariable analysis of factors associated with hospitals’ participation in health information exchange from 2014 to 2020. Multivariable logistic regression models were used with adjusted odds ratios and 95% CIs calculated from standard errors clustered at the hospital level and weighted by hospital bed size.

After controlling for other hospital factors, teaching hospitals (adjusted odds ratio [AOR] 3.7, 95% CI 1.0-13.3), rural referral hospitals (AOR 8.0, 95% CI 1.2-53.4), and system-affiliated hospitals (AOR 6.6, 95% CI 3.2-13.7) had higher odds of participating in HIE, whereas CAHs (AOR 0.1, 95% CI 0.02-0.6) were less likely to participate in HIE. Hospitals with greater ratios of Medicare inpatient days to total inpatient days or Medicaid inpatient days to total inpatient days also reported higher odds of HIE participation (Figure 4).

## Discussion

### Principal Findings

In 2014, the Office of the National Coordinator for Health Information Technology launched a 10-year road map for the United States to achieve interoperability of EHRs by 2024. In this longitudinal observational study of 69 SC hospitals, we found that federal and state efforts to build an HIE system across all hospitals have been successful in SC, with over 80% of SC hospitals participating in HIE as of 2020; however, there is still a long way to go to achieve Meaningful Use Stage 3 for improved health outcomes. We also found teaching hospitals, system-affiliated hospitals, rural referral hospitals, and hospitals with greater ratios of Medicare or Medicaid inpatient days to total inpatient days were more likely to participate in HIE, while CAHs were less likely to participate in HIE. Yet, key barriers for effective use of HIE for patient care coordination and improved health outcomes remain, including technical, data management, and legal issues. These findings highlight the need to ensure the accuracy of patient information, standardization of health information across various systems, and training of privacy and security regulations to optimize HIE use for data exchange and patient care transition.

As of 2020, although over 80% of SC hospitals participated in HIE, our findings suggest that rural hospitals experienced no greater probability of HIE participation. This might be caused by multiple barriers to HIE implementation, including challenges with reporting, workflow, and technology capacity (eg, availability of adequate broadband), even with generous financial incentives [21]. Our findings also indicate that CAHs were less likely to participate in HIE than prospective payment system hospitals, leading to delayed development of EHR interfaces by private hospitals at the early stage of HIE expansion. This result is consistent with the findings of a previous study that CAHs with EHRs still struggle to keep up with other advanced functions, such as patient engagement and clinical data analytics, as of 2018 [19]; these hospitals face skyrocketing costs and a lack of technical expertise when developing HIE interfaces, making HIE adoption a prohibitive move [19,25]. Given that CAHs were less likely to adopt advanced functions for electronic records [33], challenges for CAHs to participate and use HIE may be further exacerbated. Moreover, hospitals with a greater ratio of Medicaid or Medicare inpatient days to total inpatient days reported higher probabilities of HIE participation. This result was consistent with prior research, indicating that broader changes in the payment of care may encourage health care providers to use EHRs [34].

Many barriers to HIE implementation and use exist. Despite the HIE functionality, we found that much critical information was missing or hard to find. For example, over two-thirds of HIE-active hospitals reported difficulties locating providers’ addresses to send and receive information or finding relevant patient health information. Even with information availability, some had difficulties matching or identifying the correct patients between health care or EHR systems. On top of the information discontinuity, technical challenges in exchanging across different vendor platforms and the fact that some providers do not typically exchange patient data with outside providers further fuel the structural obstacles to the exchange and interoperability of patient health information across hospitals. In the era of telehealth and HIE across clinicians and hospitals for optimal quality of care [35,36], it is striking to find over half of SC hospitals reported barriers to the exchange of patient health information across different vendor platforms. Over half of providers do not typically exchange patient data, which may be because increasing data volumes and types is difficult and labor-intensive to match [37,38]. These barriers raise concerns of approaches at systematic and provider levels to improve the quality of health information, the health information system, and telehealth services.

Lacking provider addresses and difficulties in finding relevant patient health information were the main barriers to exchange in SC hospitals in 2014, suggesting that compatibility issues between EHRs and HIE systems are a significant systematic barrier [39]. Technical integration across distinct EHR platforms can be a real challenge given that Stage 1 of Meaningful Use did not require usability, data integrity, harmonization, and terminology mapping and matching among certified EHR systems. Moreover, the inability to get vendor support to address specific needs within hospitals limits the use of HIE for patient care transitions. Vendor issues are inextricably linked to EHR utility in many cases. Hospitals often need to use additional tools (eg, web-based data entry platforms) to retrieve specific data from their EHRs, limiting interoperability and optimization of HIE among hospitals.

Ensuring usability of information in clinical decision-making and the perception of accomplishing goals is critical for ensuring the sustainability of HIE use across hospitals facing barriers to exchange and interoperability [40]. However, in 2020, nearly 40% of hospitals reported that many recipients of electronic care summaries found that the information was not useful, and about 30% of providers could not exchange health information due to privacy laws or additional costs, which have posed obstacles to the use of HIE among health care providers. Given that physician-level variation in EHR documents can impede effective use of patient health information [41], the use of HIE may be lower when patients are unfamiliar with the health care provider [42]. This result might also be related to the fact that health care providers are more concerned about economic and competitive risks than perceived benefits [14,27]; therefore, they may complain about these issues [27], which might hinder the use of HIE in SC hospitals and discourage opportunities for improving patient care and outcomes [40]. Additionally, a previous study revealed that state and federal health information privacy laws, beyond economic and technical barriers, have been a significant obstacle to expanding HIE [27]. As the exchange of patient health information is a trust-related behavior, providers’ perceived trust in HIE’s technical capabilities, skills, and benefits warrants improvement [43]. Use of HIE by the “marquee user” should aim to attract more users to the platform and eliminate these barriers [16]. These findings suggest that the existing infrastructure may not consider the unique needs of patients who often access multiple health care service sites across multiple geographies. As many nontechnological factors may hinder the effective use of HIE [44], more efforts beyond addressing workflow and technical issues are needed to make the HIE sustainable [45]. This result also raises concerns about the full connectivity of the statewide framework, including connecting unaffiliated ambulatory care practices [46] and skilled nursing facilities [47].

### Limitations

This study has some limitations. First, the AHA Annual Surveys IT Supplement asked about hospital-wide use of HIE via a single item, and the hospitals did not report the extent of use. About 60% of hospitals responded to items on the barriers to exchange and interoperability. Assuming that SC hospitals without HIE tend not to respond to items on the barriers to exchange and interoperability, the current state-level barriers to exchange and interoperability may be underestimated. Second, we did not have data about local HIE needs. Third, the sample hospitals that reported actively participating in HIE may not have exercised HIE to its full potential. Fourth, the AHA Annual Surveys IT Supplement did not collect comprehensive vendor-specific information, and heterogeneity of vendors was not accounted for in this study. Nevertheless, our findings have revealed vendor-specific barriers to exchange and interoperability. Future research is warranted to examine how often HIE has been used across patients or patient visits/admissions and to assess key factors facilitating the optimal use of HIE.

### Conclusions

Nearly all hospitals in SC participate in HIE. However, barriers to exchange and interoperability remain, including technical, data management, user training and support, and legal issues, highlighting a crucial missed opportunity to improve health outcomes. These findings imply the need to incentivize optimization of HIE and seamless patient information exchange across facilities by facilitating and implementing standardization of health information across various HIE systems and by training HIE stakeholders about privacy and security regulations to ensure smooth, safe, and secure patient care transitions. Future policies and efforts should promote collaborations with vendors to reduce platform compatibility issues and increase user engagement and technical training and support to facilitate effective, accurate, and efficient exchange of patient health information.

## Appendix

Multimedia Appendix 1 Proportion of rural and urban hospitals participating in a health information exchange or health information organization from 2014 to 2020.

## Abbreviations

AOR: adjusted odds ratio
AHA: American Hospital Association
CAH: Critical Access Hospital
EHR: electronic health record
HIE: health information exchange
HIO: health information organization
HITECH: Health Information Technology for Economic and Clinical Health
SC: South Carolina
