# Supplementary figures and images for: Successes and Barriers of Health Information Exchange Participation Across Hospitals in South Carolina From 2014 to 2020: Longitudinal Observational Study
Source: JMIR Med Inform. 2023 Sep 28;11:e40959. doi: 10.2196/40959 (PMC10570901; doi:10.2196/40959)

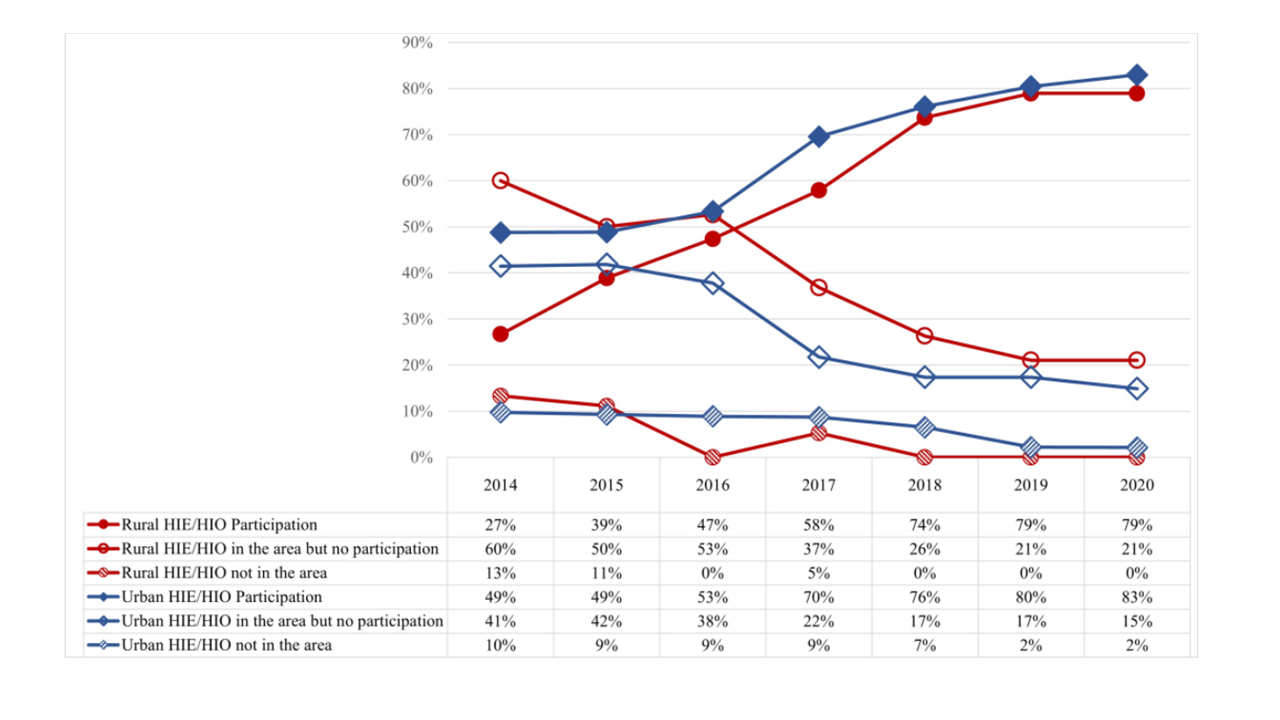

Supplement: Multimedia Appendix 1 [file medinform_v11i1e40959_app1.png]
